# Melatonin Protects Neural Stem Cells Against Tri-Ortho-Cresyl Phosphate-Induced Autophagy

**DOI:** 10.3389/fnmol.2020.00025

**Published:** 2020-03-06

**Authors:** Chang Liu, Wenjuan Zhou, Zhaopei Li, Jun Ren, Xian Li, Shan Li, Qian Liu, Fuyong Song, Aijun Hao, Fuwu Wang

**Affiliations:** ^1^Key Laboratory for Experimental Teratology of Ministry of Education, Shandong Key Laboratory of Mental Disorders, Department of Anatomy and Histoembryology, School of Basic Medical Sciences, Shandong University, Jinan, China; ^2^Department of Oncology, Shandong Provincial Hospital Affiliated to Shandong University, Jinan, China; ^3^Institute of Toxicology, School of Public Health, Shandong University, Jinan, China

**Keywords:** tri-ortho-cresyl phosphate, neural stem cells, autophagy, melatonin, cytotoxicity

## Abstract

Tri-ortho-cresyl phosphate (TOCP) is an extensively used organophosphate in industry. It has been proven to lead to toxicity in different organ systems, especially in the nervous system. Neural stem cells (NSCs) play important roles in both embryonic and adult nervous systems. However, whether TOCP induces cytotoxicity in embryonic NSCs remains unclear. In this study, mouse NSCs were exposed to different concentrations of TOCP for 24 h. The results showed that TOCP led to impaired proliferation of NSCs and induced the autophagy of NSCs by increasing the generation of intracellular reactive oxygen species (ROS) and decreasing the phosphorylation of extracellular regulated protein kinase (ERK1/2). Melatonin has been reported to exert neuroprotective effects *via* various mechanisms. Therefore, we further investigate whether melatonin has potential protective effects against TOCP-induced cytotoxicity on NSCs. Our data showed that melatonin pretreatment attenuated TOCP-induced autophagy by suppressing oxidative stress and restoring ERK1/2 phosphorylation consistently. Taken together, the results indicated that TOCP induced the autophagy in mouse NSCs, and melatonin may effectively protect NSCs against TOCP-induced autophagy.

## Highlights

– Tri-ortho-cresyl phosphate (TOCP) suppressed neural stem cell (NSC) proliferation but not neural differentiation and cell apoptosis.
– Tri-ortho-cresyl phosphate induced the autophagy of NSCs by increasing reactive oxygen species (ROS) level and decreasing phosphorylated extracellular regulated protein kinase.
– Melatonin could effectively protect NSCs against TOCP-induced autophagy.

## Introduction

Tricresyl phosphate (TCP), an extensively used organophosphate compound, widely acts as plasticizers, flame retardants, components of jet engine oil, and phosphorus-containing pesticides in industry, even in chemical fibers, textiles, and leather. TOCP is the most toxic compound among three isomers of TCP and has caused several poisoning incidents (Wolkoff et al., 2016; Lorke et al., 2017). TOCP has already been demonstrated to induce neurotoxicity, immunotoxicity (Brinkerhoff et al., 1981), and reproductive toxicity such as inhibition of spermatogonial stem cells and ovarian failure (Xu et al., 2016) in animals. Embryonic neurodevelopment plays an important role in the development of adult brain function. Among that, neural stem cells (NSCs) have the capability of self-renewal and differentiation for brain development. NSCs give rise to new neurons and glia throughout life and play key roles in both embryonic and adult nervous system (Gage and Temple, 2013). A number of studies proved that TOCP could damage structures and related functions of neurons (Chen et al., 2013; Song et al., 2015). Especially, TOCP was reported to cause severe and irreversible delayed neuropathy, namely, organophosphate-induced delayed neurotoxicity (OPIDN) in humans and sensitive animals. However, the effect of TOCP in NSCs in the developing brain remains unclear. Therefore, we investigated whether TOCP induced cytotoxicity on NSCs as well as its mechanisms.

Autophagy is an essential protein degeneration pathway to maintain intracellular homeostasis in eukaryotic cells. Under starving or stress conditions, autophagy is initiated to eliminate long-lived protein and damaged organelles by isolating these cytoplasmic components in autophagosomes and delivering them to lysosomes for degradation (Yorimitsu and Klionsky, 2005; He and Klionsky, 2009). Increasing evidence has shown that autophagy is involved in Alzheimer disease, Parkinson disease, Huntington disease, and other neurodegenerative diseases (Frake et al., 2015). Although autophagy was considered as a protective process when cells were faced with nutrition hunger or stress stimulation, some studies suggested that autophagy could also cause cell death, which is known as autophagic cell death (Levine and Kroemer, 2008). Recent studies showed that the neurotoxicity of TOCP on human neuroblastoma SH-SY5Y cells, as well as its reproductive toxicity on rat spermatogonial stem cells, was related to autophagy (Liu et al., 2015; Xu et al., 2017). In addition, TOCP was also reported to induce the autophagy of mouse Leydig TM3 cells *in vitro* *via* activating the oxidative stress (Liu et al., 2016). However, whether TOCP induces autophagy in NSCs and its potential mechanisms are unclear.

Melatonin is an endogenous hormone mainly secreted from the pineal gland in mammal brain (Reiter, 1991), which plays a critical role in physical activities including regulation of circadian rhythms and reproductive and neuroendocrine actions (Dubocovich, 2007; Hardeland, 2008). Recently, several studies have shown that melatonin could significantly decrease the production of ROS under various conditions acting as an endogenous free radical scavenger and antioxidant (Wang et al., 2013; Brazão et al., 2015; Torres et al., 2015). In addition, melatonin could also protect various cells through modulating multiple signaling pathways (Janjetovic et al., 2014; Yu et al., 2014; Lamont et al., 2015). For example, melatonin has been demonstrated to protect NSCs under pathological conditions by inhibiting the production of ROS and regulating the expression of signaling pathway proteins (Fu et al., 2011; Song et al., 2015). Moreover, melatonin was reported to be involved in the cell protection by inhibiting the autophagy (Pi et al., 2015; Yoo et al., 2016). However, whether melatonin has a protective effect on TOCP-treated NSCs is still unknown.

Therefore, the purpose of the current study was to explore the effects of TOCP on NSCs, the protective role of melatonin on the TOCP-induced toxicity of NSCs, and the underlying molecular mechanisms. We report here that melatonin pretreatment significantly attenuated TOCP-induced autophagy of NSCs, at least in part, by suppressing oxidative stress and consistently restoring extracellular regulated protein kinase (ERK1/2) signaling pathway.

## Materials and Methods

### Materials

TOCP (purity >99%) was obtained from BDH Chemicals Company Limited (Poole, UK). Dulbecco modified Eagle medium (DMEM)/F12 (1:1) medium and B27 supplement were purchased from Gibco BRL (Caithersburg, MD, USA). Basic fibroblast growth factor (bFGF) was purchased from R&D Systems, Minneapolis, MN, USA. Bafilomycin A_1_ (Baf A_1_), N-acetylcysteine (NAC), melatonin, and 4,5-dimethyl-2-thiazolyl)-2,5-diphenyl-2-H-tetrazolium bromide (MTT) were purchased from Sigma-Aldrich (St. Louis, MO, USA). Cell-Light™ 5-ethynyl-2′-deoxyuridine (EdU) Apollo^®^488 *in vitro* Imaging Kit (100T) was purchased from RiboBio Company Limited (Guangzhou, China). Annexin V–fluorescein isothiocyanate (FITC)/propidium iodide (PI) Apoptosis Detection Kit was purchased from Abcam, Cambridge, MA, USA. The primary antibodies: rabbit anti–light chain 3 beta (LC3B), rabbit anti–neuronal class III β-tubulin (Tuj-1), and rabbit anti–glial fibrillary acidic protein (GFAP) were purchased from Cell Signaling Technology, Danvers, MA, USA. Monoclonal anti–β-actin, goat anti–rabbit immunoglobulin G (IgG), and anti–mouse IgG were purchased from Sigma-Aldrich (St. Louis, MO, USA). Bicinchoninic acid assay protein assay kit was purchased from Pierce Biotechnology Inc., Rockford, IL, USA. The 2′,7′-dichlorodihydro-fluorescein diacetate (H_2_DCFDA) and dihydroethidium (DHE) were purchased from Molecular Probes, Eugene, OR, USA.

### Cell Culture

The primary NSCs were isolated and cultured according to a previously described method with minor modifications (Fu et al., 2011; Chen et al., 2016). NSCs were initially derived from embryonic brain of Kunming mice at embryonic day 12.5. The entire cerebrum was separated from embryonic brain and then was placed into ice-cold Hanks balanced salt solution. Following mechanical separation, cells were centrifuged, resuspended, and incubated with DMEM/F12(1:1) medium plus 2% B27, 20 ng/ml bFGF, 100 U/ml penicillin, and 100 mg/ml streptomycin at 37°C in a humidified atmosphere of 5% CO_2_. The culture medium was replaced, and NSCs were mechanically separated again every 2 days. Animal care and treatment complied with the National Institutes of Health Guide for the Care and Use of Laboratory Animals, and the animal experiments were approved by the Institutional Animal Care and Use Committees of Shandong University (No. 201402020).

### Cell Treatment

NSCs at two to four passages were collected by centrifuging at 600 *g* for 5 min and resuspended in medium with 0–100 μM TOCP. Meanwhile, 10 nM Baf A_1_, 5 μM NAC, 50 μM PD98059 (specific inhibitor of ERK), or 40 μM melatonin was added into the medium before TOCP treatment, respectively. The cells were then cultivated for another 24 h and were processed for further research.

### MTT Assay

The cell viability of NSCs was detected by MTT assay. The NSCs were seeded into 96-well plates (Corning Inc., Corning, NY, USA) preincubated with poly-L-lysine (PLL). The density of cells was 5 × 10^4^ cells/well with 200 μl culture medium per well. After the cells were adherent, the NSCs were treated with 0–100 μM TOCP for 24 h in the presence or absence of 10 nM Baf A_1_, 5 μM NAC, and 40 μM melatonin, respectively. Then, 20 μl MTT (0.5 mg/ml) was added into each well for another 4 h, and the culture medium was replaced by 150 μl DMSO per well. The absorbance was determined by spectrophotometer at 490 nm.

### EdU Assay

Cell proliferation activity was determined by EdU assay according to the manufacturer’s instructions. Briefly, the NSCs were planted on glass cover slips pretreated with PLL and were exposed to 0–100 μM TOCP for 24 h. Cells were incubated with EdU reagent at room temperature for 1 h and washed with phosphate-buffered saline three times. The photographs were taken by a fluorescence microscope (IX71; Olympus, Tokyo, Japan).

### Western Blot Analysis

The differently treated NSCs were collected, washed with cold phosphate-buffered saline, and lysed with cold RIPA buffer. After incubation at 4°C for 30 min, the samples were centrifuged at 11,000 *g* for 10 min at 4°C. The protein concentration was detected by a BCA protein assay kit (Pierce Biotechnology Inc., Rockford, IL, USA). Samples were loaded on to sodium dodecyl sulfate–polyacrylamide gel electrophoresis, separated on 8% to 15% gradient gels, and electrophoretically transferred to polyvinylidene fluoride membranes. After transfer, the membranes were blocked with 5% fat-free milk for 90 min at room temperature. The membranes were then incubated with specific primary antibodies (1:1,000), at 4°C overnight, respectively. After three washes with tris-buffered saline tween-20 (TBST) buffer, the membranes were incubated with the corresponding secondary antibody (1:10,000) at room temperature for 1 h, respectively. Monoclonal anti–β-actin acts as an internal control. The membranes were finally incubated with ECL reagents, and immunoreactive bands were detected using the Image-Pro Plus 6.0 software (Media Cybernetics, Inc., Rockville, MD, USA).

### Annexin V–FITC/PI Staining Assay

The NSCs with different treatments were collected in a single-cell suspension in 500 μl binding buffer per sample at 37°C, and 5 μl annexin V–FITC and 5 μl PI were added into the medium, respectively, according to the manufacturer’s instructions. The results were analyzed by flow cytometry within 1 h.

### Transmission Electron Microscopy Analysis

After treatment with 0–100 μM TOCP for 24 h, NSCs were harvested and centrifuged. The supernatant was replaced by cold 2.5% glutaraldehyde for 2 h and osmium tetroxide (OsO4) for 1 h. After the protocols of dehydration and embedding, double stain was administered on ultrathin sections (60 nM) with uranyl acetate and lead citrate, and the observation was taken by transmission electron microscope (TEM).

### ROS Assay

NSCs (1 × 10^6^ cells/well) were cultured in six-well culture plates pretreated with PLL. After various treatments, the cells were incubated with 10 μM H_2_DCFDA and 2 μM DHE for 30 min at 37°C, respectively. Following counterstaining by 4′, 6-diamidino-2-phenylindole (DAPI), the images were taken with fluorescence microscope (IX71; Olympus). Meanwhile, the fluorescence intensity of the samples was determined by flow cytometry, respectively.

### Statistical Analysis

The data are expressed as mean ± SD of at least three independent experiments. The values were analyzed by one-way analysis of variance with SPSS 13.0 software (International Business Machines Corporation, Armonk, NY, USA). *P* < 0.05 was considered statistically significant.

## Results

### Effect of TOCP on the Cell Viability of Mouse NSCs

**Figure 1:**
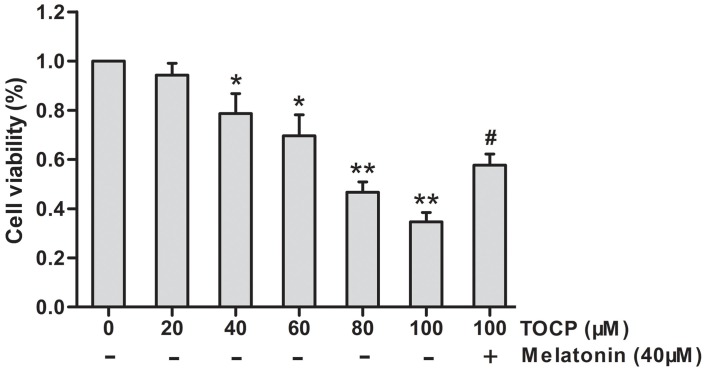
Effects of tri-ortho-cresyl phosphate (TOCP) on the cell viability of mouse neural stem cells (NSCs). NSCs were treated with different concentrations of TOCP (0–100 μM) with or without pretreatment by 40 μM melatonin for 30 min. The cell viability was detected by MTT assay. Data are expressed as mean ± SD of at least three independent experiments. *P < 0.05, **P < 0.01 vs. control; ^#^*P* < 0.05 vs. 100 μM TOCP alone.

First, the effect of different concentrations of TOCP (0–100 μM) on NSC cell viability was investigated by MTT assay. As shown in Figure 1, TOCP decreased the cell viability of NSCs in a dose-dependent manner. According to the MTT results, two concentrations of TOCP (40 and 100 μM) were used in the following study to detect the effect on NSCs.

### Effect of TOCP on Proliferation, Differentiation, and Cell Apoptosis of Mouse NSCs

**Figure 2:**
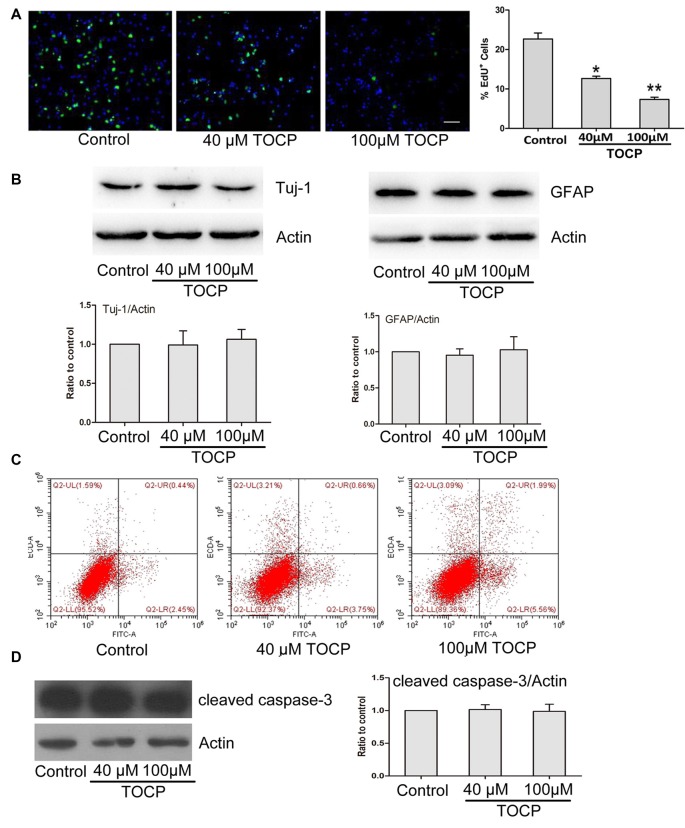
Effects of TOCP on proliferation, differentiation, and cell apoptosis of mouse NSCs. NSCs were treated with TOCP (40 and 100 μM, respectively). **(A)** The cell proliferation was determined by EdU assay. Scale bar, 50 μm. **(B)** The neural differentiation-related proteins Tuj-1 (a neuronal marker) and glial fibrillary acidic protein (GFAP; an astrocyte marker) were detected by Western blot. **(C)** The cell apoptosis of TOCP-treated NSCs was assessed using annexin V–FITC/PI double staining by flow cytometry. **(D)** The apoptosis-related protein, cleaved caspase-3 was examined by Western blot. Data are presented as mean ± SD of at least three independent experiments. *P < 0.05, **P < 0.01 vs. control.

To further assess the characteristics of NSCs under TOCP exposure condition, a series of experiments were conducted. First, the EdU assay was performed to detect the effect of TOCP (40 and 100 μM for 24 h) on the proliferation of NSCs, and the results showed that TOCP treatment significantly decreased the percentage of EdU-positive cells compared with the control (Figure 2A, *P* < 0.05 and *P* < 0.01, 40 and 100 μM TOCP, respectively). The results suggest that the proliferation of NSCs was suppressed under TOCP exposure condition in the present study.

Then, the effect of TOCP on the neural differentiation of NSCs was observed by detecting the expression of differentiation-associated proteins. Western blot revealed that TOCP did not significantly change the protein expression levels of Tuj-1 (a neuronal marker) and GFAP (an astrocyte marker) in NSCs (Figure 2B), which implied that TOCP had no significant effects on the neural differentiation of NSCs.

Finally, the cell apoptosis of NSCs induced by TOCP was examined using annexin V–FITC/PI double-staining method. Annexin V and PI can be used to distinguish early and late apoptosis cells and dead cells. The flow cytometry results displayed that both 40 and 100 μM TOCP did not significantly change the apoptotic or necrotic ratio of NSCs compared with the control, and there was also no significant difference of apoptotic ratio between 40 and 100 μM TOCP groups (Figure 2C). In addition, similar results were obtained by detecting the expression of apoptosis-associated protein, cleaved caspase-3 (Figure 2D). Our data suggest that TOCP also did not significantly affect the cell apoptosis of NSCs in the concentration ranges of the present study.

### Melatonin Attenuates TOCP-Induced Autophagy in Mouse NSCs

**Figure 3:**
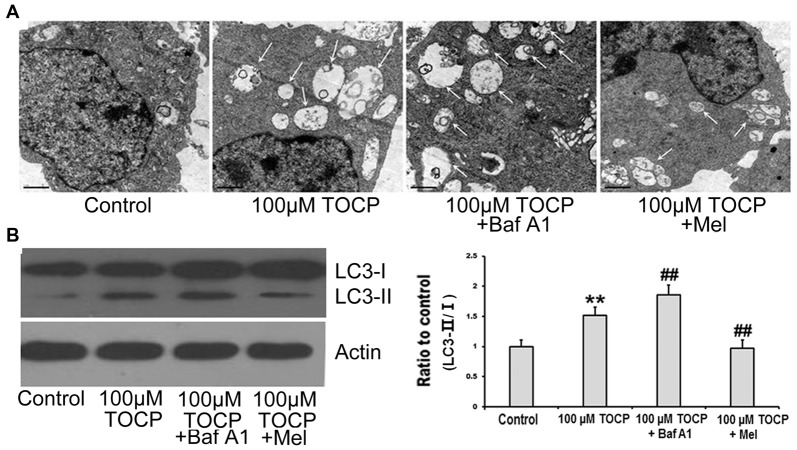
Melatonin attenuates TOCP-induced autophagy in mouse NSCs. NSCs were treated with 100 μM TOCP with or without the pretreatment of 10 nM bafilomycin A_1_ (Baf A_1_) and 40 μM melatonin, respectively. **(A)** The formation of autophagosomes was detected by transmission electron microscope (TEM). Scale bar, 1 μm. **(B)** Autophagy-related protein LC3B was analyzed by Western blot. Data are expressed as mean ± SD of at least three independent experiments. **P < 0.01 vs. control, ^##^*P* < 0.01 vs. 100 μM TOCP.

To explore whether autophagy is involved in the toxicity of TOCP on NSCs, the formation of autophagosomes was first detected by TEM. The results showed that the number of autophagosomes significantly increased in NSCs treated with 100 μM TOCP for 24 h (Figure 3A). Meanwhile, autophagy-related protein LC3B was also analyzed by Western blot. As shown in Figure 3B, 100 μM TOCP markedly increased the ratio of LC3-II to LC3-I in NSCs, which was consistent with the results of TEM.

To further determine whether the increase of autophagosomes was due to the enhancement of autophagy activity or the block of autophagy flux in TOCP-treated NSCs, 10 nM Baf A_1_ (a specific inhibitor of autophagy at a latest stage) was preadministered for 30 min into the medium before 100 μM TOCP exposure for 24 h. The results showed that Baf A_1_ pretreatment further increased both the number of autophagosomes and the ratio of LC3-II to LC3-I compared to TOCP treatment alone (Figures 3A,B), which implied that TOCP indeed enhanced the complete autophagy flux in NSCs.

To investigate the effect of melatonin on TOCP-treated NSCs, we first analyzed the cell viability of NSCs by MTT assay. The results showed that melatonin (40 μM) pretreatment for 30 min before TOCP exposure significantly restored the cell viability of NSCs compared with 100 μM TOCP treatment alone (Figure 1, *P* < 0.05). Then, the protective effects of melatonin on TOCP-induced autophagy were detected. The TEM results displayed that melatonin pretreatment could significantly reduce the number of autophagosomes (Figure 3A). Western blot also showed that melatonin markedly decreased the ratio of LC3-II to LC3-I (Figure 3B). The results implied that melatonin could inhibit the autophagy induced by TOCP in NSCs.

### Melatonin Decreases the Production of ROS Induced by TOCP in Mouse NSCs

**Figure 4:**
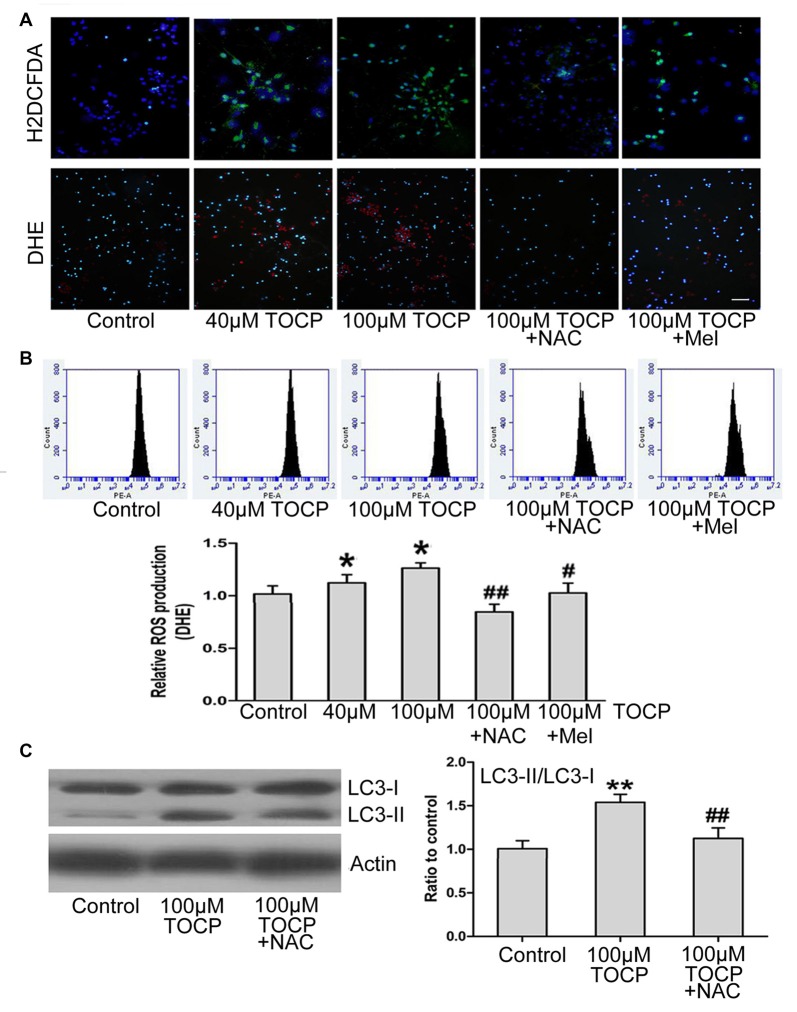
Melatonin decreases the production of reactive oxygen species (ROS) induced by TOCP in mouse NSCs. NSCs were exposed to different concentrations of TOCP (40 or 100 μM) with or without 5 μM NAC and 40 μM melatonin, respectively, before TOCP exposure. **(A)** The intracelllular ROS level was examined by H2DCFDA (green) and DHE (red) assay. The cells were then counterstained by DAPI (blue). Scale bar, 50 μm. **(B)** The results of DHE staining were also observed by flow cytometry. **(C)** NSCs were pretreated with 5 μM NAC before 100 μM TOCP exposure, and autophagy-related protein, LC3, was analyzed by Western blot. Data are expressed as mean ± SD of three individual experiments. *P < 0.05, **P < 0.01 vs. control, ^#^*P* < 0.05, ^##^*P* < 0.01 vs. 100 μM TOCP alone.

Previous studies have shown that TOCP could trigger the oxidative stress and autophagy (Liu et al., 2016). To determine whether oxidative stress is relevant to TOCP-treated NSCs, the intracellular ROS level was examined by H_2_DCFDA and DHE assay, respectively (Figure 4A). Fluorescence photomicrography and flow cytometry analysis showed that TOCP significantly increased the intracellular ROS production in NSCs (Figures 4A,B). Then, *N*-acetyl-L-cysteine (NAC, an inhibitor of oxidative stress, 5 μM) was added into the medium for 30 min before TOCP exposure. The results showed that NAC pretreatment markedly suppressed the ROS level in NSCs (Figures 4A,B) and reduced the ratio of LC3-II to LC3-I in NSCs (Figure 4C), which suggested that TOCP induced autophagy by elevating the oxidative level in NSCs. Finally, melatonin was added into the medium for 30 min before TOCP exposure, and the results showed that melatonin significantly inhibited the intracellular ROS level in NSCs (Figures 4A,B). These results suggested that melatonin could inhibit the TOCP-induced autophagy in NSCs through suppressing the oxidative stress.

### Melatonin Activates ERK1/2 Signaling Pathway Suppressed by TOCP in Mouse NSCs

**Figure 5:**
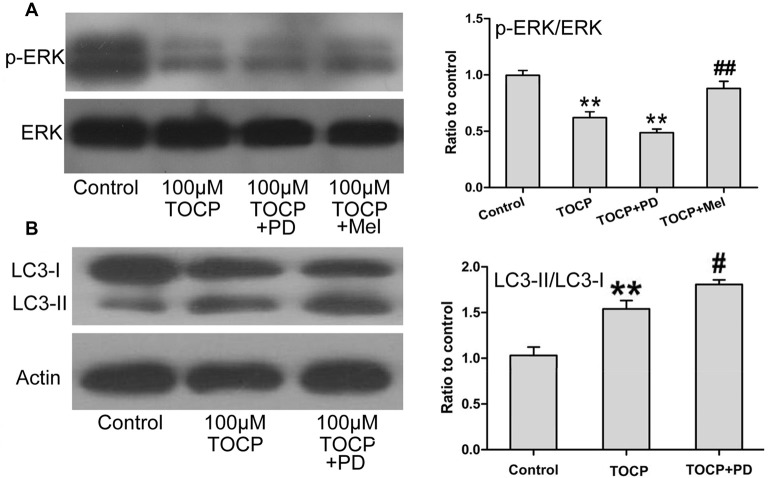
Melatonin activates ERK1/2 signaling pathway suppressed by TOCP in mouse NSCs. NSCs were treated with 100 μM TOCP in the presence or absence of 50 μM PD98059 (a specific ERK1/2 inhibitor) and 40 μM melatonin, respectively. **(A)** The expression of p-ERK in TOCP-treated NSCs was examined by Western blot. **(B)** Autophagy-related protein, LC3, was analyzed by Western blot. Data are expressed as mean ± SD of at least three independent experiments. **P < 0.01 vs. control, ^#^*P* < 0.05, ^##^*P* < 0.01 vs. 100 μM TOCP alone.

Several studies proved that ERK1/2 signaling protein participates in the autophagy of several types of cells (Jo et al., 2014; Huang et al., 2015). To further determine whether ERK1/2 signaling protein is involved in TOCP-induced autophagy of NSCs, the phosphorylation of ERK1/2 (p-ERK) was examined in NSCs treated with 100 μM TOCP for 24 h. As shown in Figure 5A, TOCP significantly decreased the expression of p-ERK (*P* < 0.01). Pretreatment with 50 μM PD98059 (specific ERK1/2 inhibitor) for 30 min significantly reduced the p-ERK level and increased the ratio of LC3-II to LC3-I (Figures 5A,B). The results suggested that TOCP may also induce the autophagy of NSCs by inhibiting the phosphorylation of ERK1/2 protein. Melatonin pretreatment significantly increased the p-ERK1/2 level compared with the TOCP treatment alone (Figure 5A). Furthermore, melatonin treatment also significantly decreased the ratio of LC3-II to LC3-I in NSCs (Figure 3B). These results suggested that melatonin could suppress the autophagy in NSCs treated with TOCP, at least partially, by restoring the phosphorylation of ERK1/2 protein.

## Discussion

Increasing studies have shown that TOCP induces the autophagy in many types of cells (Long et al., 2014; Song et al., 2014; Liu et al., 2016). However, it is still unclear whether TOCP has an effect on NSCs. The major finding of the present study was that TOCP induced the autophagy in NSCs and decreased the cell proliferation of NSCs, whereas melatonin protected the NSCs against TOCP-induced autophagy through suppressing the oxidative stress and activating the ERK1/2 signaling pathway.

TOCP has been extensively used in industry serving as pesticides, plasticizers, lubricants, additives, and chemical warfare agents. In addition to poisoning of acute organophosphorus compounds, TOCP can also cause severe and irreversible delayed neuropathy, OPIDN, in sensitive animals and humans. Although the OPIDN incidents were first reported in the 1930’s in America, there are still various theories on the pathogenesis of OPIDN (Wolkoff et al., 2016; Lorke et al., 2017). Current clinical treatments have no specific effects, and the prognosis has been poor until now, which not only significantly influenced patient health but also caused a heavy societal burden (Abdollahi and Karami-Mohajeri, 2012; Emerick et al., 2012).

NSCs exist in both the embryonic and adult nervous system and will differentiate into neurons and glia. NSC therapy has been regarded as a potential therapy for neurological diseases such as neurodegeneration, stroke, or spinal cord lesions (Gage and Temple, 2013; Tsukamoto et al., 2013). Recent studies have shown that TOCP inhibited the formation of axon-like processes in N2a and PC12 cells (Flaskos et al., 1998) and decreased the cell viability in series of cells such as neuroblastoma SH-SY5Y cells and mouse spermatogonial stem cells (Long and Wu, 2008; Liu et al., 2016). Previous studies have focused on the neurotoxicity of TOCP to neurons, but no studies have considered whether TOCP has effects on NSCs, as well as its potential mechanisms. Here, the present study demonstrated that TOCP suppressed cell viability and inhibited the proliferation of NSCs, but it had no significant effects on the differentiation and apoptosis of NSCs. The results were similar with the previous report that TOCP markedly inhibited the viability of mouse Leydig TM3 cells and had no effects on its apoptosis (Liu et al., 2016).

Autophagy is a highly conserved intracellular catabolic progress, which eliminates damaged organelles and long-lived proteins by isolating these cytoplasmic components in autophagosomes and delivering them to lysosomes for degradation. In recent years, many standard methods were used to monitor the autophagy including detecting the formation of autophagosomes and the change of LC3-II/I ratio (Klionsky et al., 2008). Transmission electron microscopy was widely used to observe the autophagosome formation and analyze both the qualitative and quantitative change of autophagy. In the present study, the results of TEM showed that TOCP treatment remarkably increased the number of autophagosomes in NSCs. In addition, LC3 protein was also extensively used to detect the level of autophagy. LC3 is initially synthesized in an unprocessed form, proLC3, which then is transformed to a proteolytically processed form, LC3-I, and is finally modified into the phosphatidylethanolamine-conjugated form, LC3-II (Sou et al., 2006). LC3-II is considered as the marker associated with completed autophagosomes, and the ratio of LC3-II to LC3-I becomes one of the gold standards for detecting the autophagy level. The present study also demonstrated that TOCP exposure significantly enhanced the ratio of LC3-II to LC3-I in NSCs. Both the TEM and LC3 detecting results suggest that TOCP indeed induced the cell autophagy of NSCs.

It is well known that autophagy is actually a process including the formation of autophagosomes, the fusion between autophagosomes and lysosomes, and the formation of autolysosomes, which is also called autophagic flux. It was reported that the accumulation of autophagosomes may be due to the increased autophagic activity or the reduced turnover of autophagosomes (Klionsky et al., 2008). Therefore, to further explore the effects of TOCP on the autophagy of NSCs, Baf A_1_, a specific inhibitor of autophagy at the latest stage by inhibiting the fusion between autophagosomes and lysosomes, was used in the present study. Bafilomycin A_1_ pretreatment further obviously enhanced the ratio of LC3-II to LC3-I in NSCs treated with TOCP. This demonstrated that TOCP treatment increased the overall autophagic flux in NSCs, and Baf A_1_ blocked the autophagic flux, which resulted in the accumulation of LC3-II and higher ratio of LC3-II to LC3-I. The findings further proved that TOCP could induce the autophagy and enhance the autophagic flux in NSCs.

Despite the considerable advances in the biology of autophagy, the functions of autophagy are still unclear. Previous studies showed that oxidative stress could induce autophagy in both humans and animals (Filomeni et al., 2015). Both oxidative stress and autophagy pathways are all relevant mechanisms of toxicity from organophosphorus compounds treatment (Kovacic, 2003; Xu et al., 2017). Moreover, some studies showed that oxidative stress was involved in TOCP-induced autophagy of mouse Leydig TM3 cells (Liu et al., 2016). However, it is unknown whether oxidative stress plays a critical role in TOCP-induced autophagy of NSCs. The present results showed that TOCP significantly activated the oxidative stress and induced the autophagy in NSCs. Meanwhile, the treatment of specific oxidative stress inhibitor NAC markedly decreased the level of oxidative stress and reduced the autophagy. Melatonin, one of the strongest antioxidants, has been reported to be involved in regulating the autophagy induced by oxidative stress, endoplasmic reticulum stress, and mitochondria dysfunction (Fernández et al., 2015; Wang et al., 2015). The current study showed that melatonin also significantly decreased TOCP-induced oxidative stress in NSCs and the ratio of LC3-II to LC3-I. These results demonstrated that TOCP could significantly increase the level of oxidative stress in NSCs, and the higher oxidative stress level in turn induced the autophagy of NSCs; meanwhile, melatonin could protect the TOCP-treated NSCs by inhibiting oxidative stress.

In addition to oxidative stress, multiple signaling pathways including ERK1/2 signaling proved to play important roles in cell autophagy (Parzych and Klionsky, 2014). In the present study, our results showed that TOCP decreased the expression of p-ERK in NSCs, and the specific ERK1/2 inhibitor PD98059 pretreatment further decreased the expression of p-ERK and increased the ratio of LC3-II to LC3-I in TOCP-treated NSCs. Melatonin has strong antioxidant characteristics and is involved in the cell autophagy by modulating series of signaling pathways (Yoo et al., 2016; Zhang et al., 2016). The present study showed that melatonin significantly increased the expression of p-ERK in NSCs and decreased the autophagy of NSCs, which was consistent with previous results that melatonin could protect cells against pathological condition–induced autophagy through regulating the ERK1/2 signaling pathway (Yoo et al., 2016; Zhang et al., 2016).

## Conclusion

Numerous investigations have been conducted on TOCP toxicity, which include various mechanism studies such as acetyl cholinesterase inhibition, neuropathy target esterase inhibition, or γ-aminobutyric acid antagonization (Lorke et al., 2017) in the neural system (Craig and Barth, 1999; Zhang et al., 2007), immune system (Foil et al., 1980; Brinkerhoff et al., 1981), or reproductive system (Xu et al., 2016), but consensus regarding this topic has not been reached. The definite effects and potential mechanisms of TOCP on NSCs, which are essential in the nervous system, still remain to be explored and verified. This study demonstrated that TOCP induced the autophagy of NSCs and decreased cell viability and proliferation. In addition, melatonin could significantly suppress TOCP-induced autophagy of NSCs by inhibiting the oxidative stress level and activating the ERK1/2 signaling pathway and consequently restore cell viability of NSCs. Our study may contribute to forming a more comprehensive and systematic understanding of the toxicity of TOCP, especially in the nervous system, and serving as a reference for clinical treatment of TOCP poisoning cases.

## Data Availability Statement

All datasets generated for this study are included in the article.

## Ethics Statement

The animal study was reviewed and approved by Animal care and treatment complied with the National Institutes of Health Guide for the Care and Use of Laboratory Animals and the animal experiments were approved by the Institutional Animal Care and Use Committees of Shandong University (No. 201402020).

## Author Contributions

CL and WZ contributed to the conception and design of the study. ZL, JR, XL, SL and QL organized the database. FS performed the statistical analysis. CL, WZ, and AH wrote the first draft of the manuscript. FW wrote sections of the manuscript. All authors contributed to manuscript revision, read and approved the submitted version.

## Conflict of Interest

The authors declare that the research was conducted in the absence of any commercial or financial relationships that could be construed as a potential conflict of interest.
